# Supplementary material for: Altered microRNA Transcriptome in Cultured Human Liver Cells upon Infection with Ebola Virus
Source: Int J Mol Sci. 2021 Apr 6;22(7):3792. doi: 10.3390/ijms22073792 (PMC8038836; doi:10.3390/ijms22073792)
Supplement: Supplementary file 1 [file ijms-22-03792-s001.zip › Supplementary_File/D_ KEGG_Analysis_Results/16-30nt_pathway_Reston-24h-Huh7_vs_Control-24h-Huh7_up.mature_mirna_targets/hsa_EnrichmentScoreDotPlot.pdf]

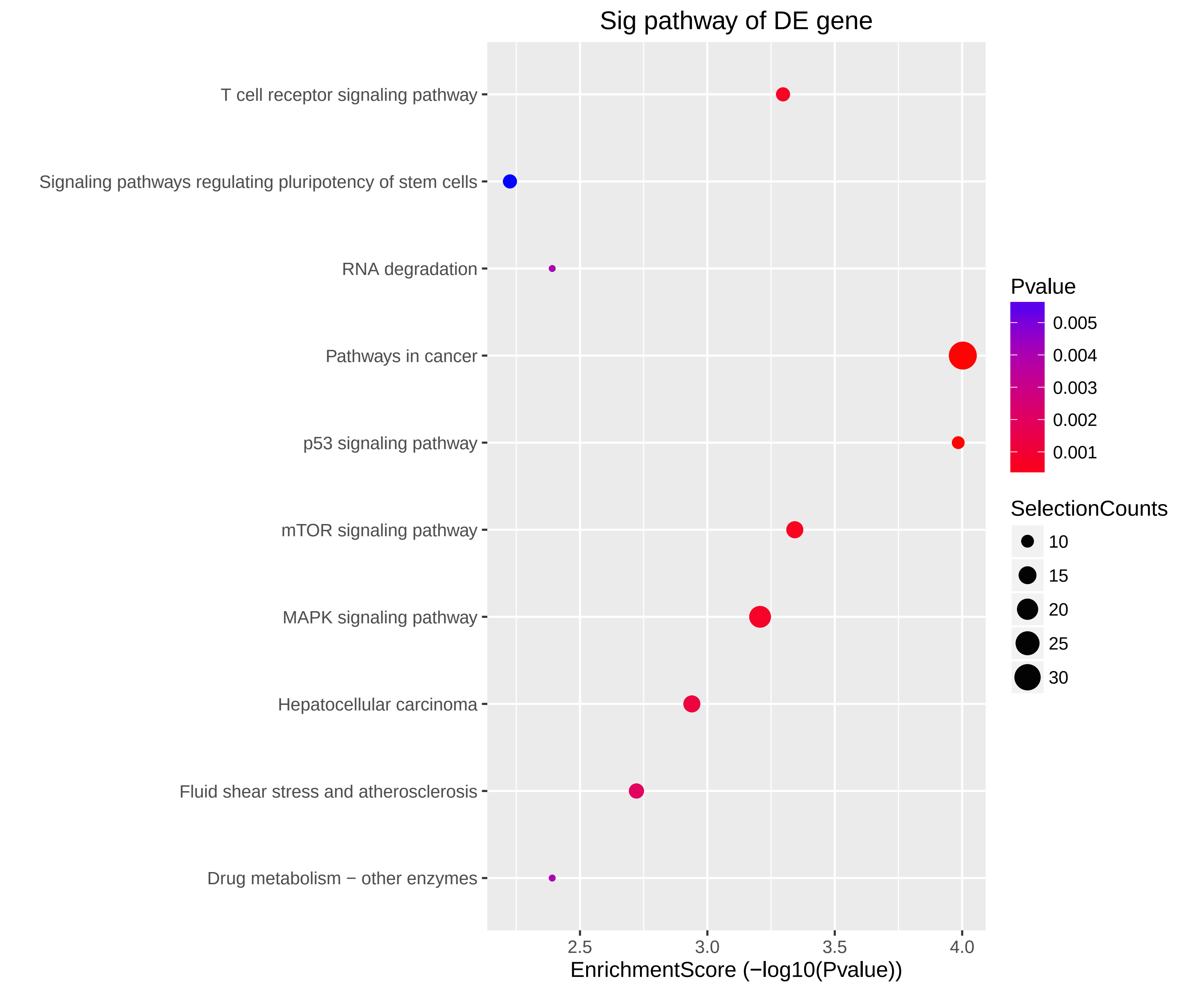
Sig pathway of DE gene
T cell receptor signaling pathway
Signaling pathways regulating pluripotency of stem cells
RNA degradation
Pathways in cancer
p53 signaling pathway
mTOR signaling pathway
MAPK signaling pathway
Hepatocellular carcinoma
Fluid shear stress and atherosclerosis
Drug metabolism − other enzymes
2.5
3.0
3.5
4.0
EnrichmentScore (−log10(Pvalue))
Pvalue
0.005
0.004
0.003
0.002
0.001
SelectionCounts
10
15
20
25
30
